# Preliminary evaluation of SaCoVLM video laryngeal mask-guided intubation in airway management for anesthetized children

**DOI:** 10.1186/s12871-023-01996-3

**Published:** 2023-02-08

**Authors:** Juan Zhi, Xiao-Ming Deng, Yan-Ming Zhang, Ling-Xin Wei, Qian-Yu Wang, Dong Yang

**Affiliations:** Department of Anesthesiology, Chinese Academy of Medical Sciences & Peking Union Medical College Plastic Surgery Hospital and Institute, 33 Ba-Da-Chu Road, Shi-Jing-Shan District, Beijing, 100144 China

**Keywords:** Laryngeal mask airway, Visual, Tracheal intubation, Airway management, Children

## Abstract

**Backgrounds:**

To preliminary evaluate the application of novel SaCoVLM video laryngeal mask -guided intubation for anesthetized children.

**Methods:**

One hundred twenty-four children with microtia (ages 5-15 years,) who required general intubation anaesthesia, were enrolled in the study. After induction of general anesthesia,guided tracheal intubation under direct vision of the SaCoVLM was performed. Our primary outcome was first-pass success rate of guided tracheal tube placement. Secondary outcome included glottic visualization grades, the first-attempt success rate of LMA placement, the time for LMA placement and time to endotracheal intubation as well as the time for LMA removal after successful intubation, the fiberoptic grade of laryngeal view, the baseline and postinduction hemodynamic parameters were also recorded,and the incidence 24 h complications after operation.

**Results:**

The first-pass success rate of guided tracheal tube placement was 91.1% (95%CI = 1.04–1.14), the status of glottic visualization was classified: grade 1 in 27cases, grade 2 in 36 cases, grade 3 in 41 cases and grade 4 in 20 cases. The first success rate of LMA placement was 92.7% (95%CI = 1.03–1.13), the time for LMA insertion was 15.7 (±9.1) s, intubation time was 30.9 (±17.6) s and withdrawl time was 24.9 (±9.3) s. The incidence of postoperative sore throat at 2 h was 29%, and 16.1% at 24 h, without dysphagia and hypoxia.

**Conclusion:**

The SaCoVLM video laryngeal mask-guided intubation is feasible in children, with a high success rate, could be a new promising device to guide intubation in airway management.

**Trial registration:**

This study was approved by the University’s Institutional Review Board and written informed consent was obtained from all subjects participating in the trial. The trial was registered prior to patient enrollment at clinicaltrials.gov (ChiCTR2200061481, http://www.chictr.org.cn. Principal investigator: Juan Zhi; Date of registration: 26/06/2022.

## Introduction

Optimal airway management is crucial in external ear reconstruction surgery because children with microtia may experience difficult laryngoscopy [1, 2]. The unique features of the paediatric airway, including a larger tongue, a larger and floppier epiglottis, a more cephalad and anteriorly located larynx and a more acute angle of the posterior pharyngeal wall to the floor of the mouth than in adults [3], may interfere with the ideal positioning of the LMA. Due to anatomical and physiological differences, the technique of endotracheal intubation is relatively more difficult in children [4]. The Difficult Airway Society (DAS) guideline for the management of the unanticipated difficult airway states that if the initial intubation attempt using laryngoscopy fails, a supraglottic airway device (SAD) should be placed to achieve oxygenation, it also recommended the use of fibreoptic-guided intubation through the SAD rather than blind attempts [5].

**Fig. 1:**
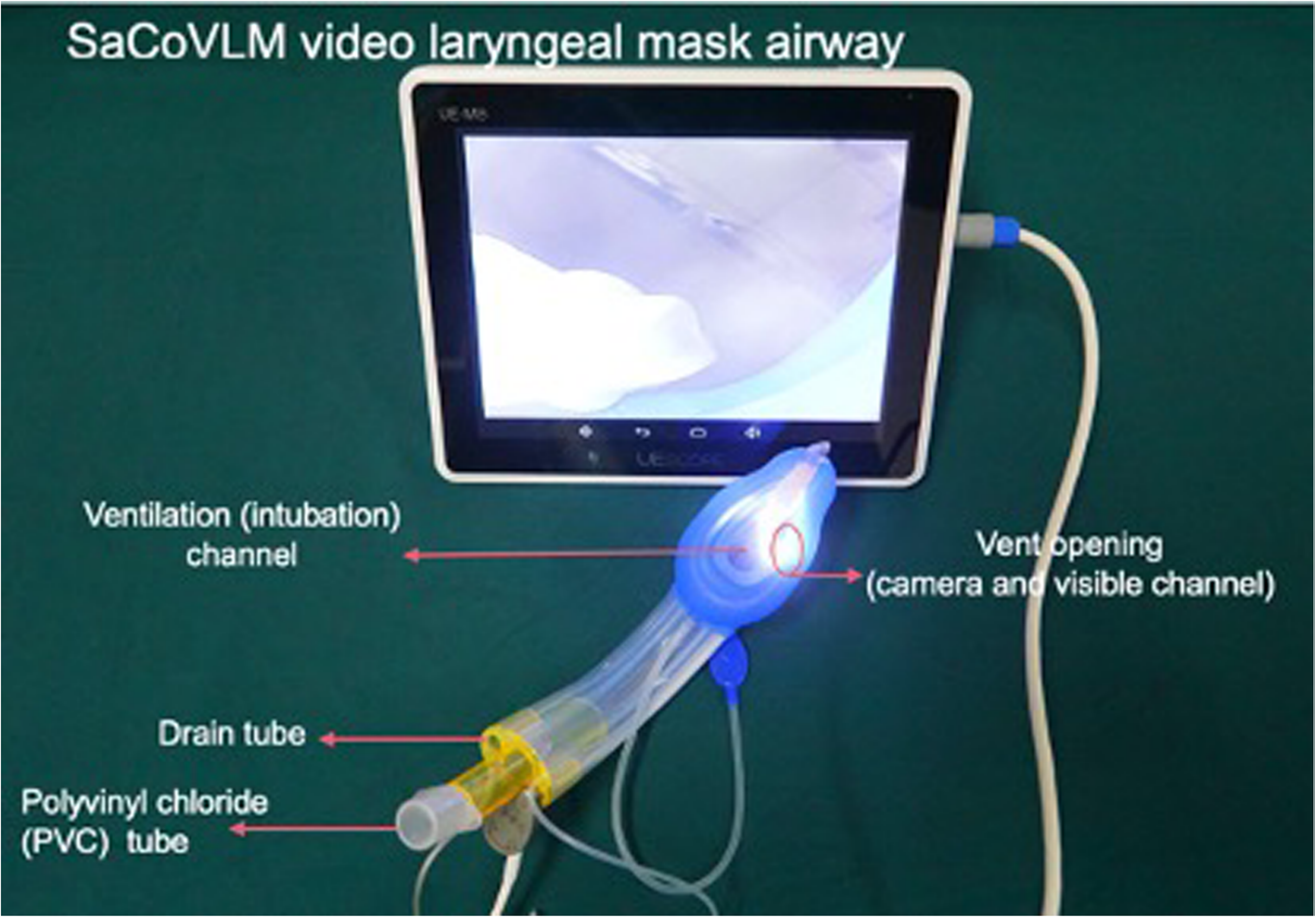
The SaCoVLM video laryngeal mask contains a ventilation (intubation) channel; a vent opening (camera and visible channel) and a drain tube channel

The SaCoVLM [6] (Safe Comfortable Video laryngeal mask, Zhejiang UE Medical Corp. Add: NO.8, YouYi Road, Baita Economic Develop Zone, Xianju, Zhejiang, China) is a newly-developed SAD combines the features of double lumen SAD and intubation type SAD. SaCoVLM has a functional sight for direct vision to guide endotracheal intubation without the need for adjuvants (Fig. 1).

Hence, our primary aim was to preliminary evaluate the application of novel SaCoVLM video laryngeal mask -guided intubation for anesthetized children and observe the visibility of SaCoVLM video laryngeal mask.

## Methods

### Subjects

This single-center study was approved by the University’s Institutional Ethical Committee (Plastic Surgery Hospital Ethical Committee No.ZX2021–12, Beijing, China) and the trial was registered prior to patient enrollment at clinicaltrials.gov (ChiCTR2200061481, http://www.chictr.org.cn. Principal investigator: Juan Zhi; Date of registration: 26/06/2022.) and we confirmed that all experiments were performed in accordance with relevant guidelines and regulations (such as the Declaration of Helsinki).

Our hospital is the largest ear reconstruction center in China with 2000 ear reconstruction surgeries per year. Between June 2022 and nov 2022, children with microtia, scheduled for elective surgery under general endotracheal anesthesia.

Inclusion criteria: we obtained written informed consent from patients’ guardians, ASA I–II, with ages between 5 and 15 years, gender unrestricted; 18 kg/m^2^ ≤ BMI ≤ 30 kg/m^2^.

Exclusion criteria: ASA III–IV, History of upper respiratory tract infection within 2 weeks; the presence of risk factors for gastric reflux or aspiration; bronchial asthma; morbid obesity (BMI>30 kg/m^2^).

The study investigators were three anaesthesiologists very well experienced in using different kinds of laryngeal mask devices.

### Preparation of SaCoVLM

The SaCoVLM includes a visual channel, a intubation channel, a gastric tube channel, a camera and connecting wires. The camera is fixed on the right side of ventral cuff,connected with the screen and inserted into the visual channel. During placement, the SaCoVLM is adjusted according to the image displayed on the screen. A recharged battery is used to provide energy. The camera was inserted into the visual channel and connected with the screen before later use. Initial size selection for the SaCoVLM was as follows: size 2.5 for patients 20–30 kg and size 3 for those 30–50 kg. However, in the pre-experimental process, it was found that size 3 laryngeal mask is too large for 30 kg children because it is difficult to insert the device under direct vision. Therefore, in the formal experiment process, we selected size 2.5 for 20-35 kg and size 3 for 35 kg–50 kg children.

Routinely check the cuff of the laryngeal mask before anesthesia induction. Use lidocaine gel to fully lubricate the back of the laryngeal mask, the inner wall of the airway tube of the laryngeal mask and the outer wall of the tracheal tube, and evacuate the cuff for use. Check the light source and clarity of the flexible intubation scope (FIS), and lubricate the stem of the flexible intubation scope.

### Preoperative preparation

All children were forbidden to drink for 6 h and fasted for 8 h before operation. The general information of the patients were asked before surgery, including age, height and weight. The modified Mallampati classification (Class I–IV), mouth opening, thyromental distance, Upper lip bite test class, Hemifacial Microsomia, Modified Mallampati (Samsoon and Young) classification [7] was assessed by an anesthesiologist ignorant of the study while the patient was sitting with the mouth wide open and the tongue protruding without phonation. Mouth opening was measured as the difference between the upper and lower incisors at the midline in centimeters using a scale. Thyromental distance [8] was measured from the thyroid cartilage to inside of the mentum with neck extended, using a tape. Peripheral venous access was initiated in the operating room, a multifunctional monitor (Datex-Ohmeda S5, General Electric, Boston, MA, USA) to monitor basic vital signs such as electrocardiogram (ECG), non-invasive blood pressure (NIBP), end-tidal-carbon dioxide (EtCO_2_) and pulse oxygen saturation was set up.

### Anesthesia and airway management

The children were preoxygenated with 100% O_2_(5L/min,5 min) before induction using a facemask and the head was placed in the neutral supine position. General anesthesia was pre-medicated with midazolam (0.02 mg/kg), sufentanil (0.25μg/kg), and. Anaesthesia was induced with propofol (2.5 mg/kg). After adequate ventilation using mask ventilation, rocuronium (0.6 mg/kg) was administered for muscle relaxation. Adequate anesthetic depth was confirmed by the disappearance of the eyelash reflex when the jaw was completely relaxed [9]. Both devices were placed using a standard midline insertion technique (the anesthetist held the distal end of the ventilation channel and let the laryngeal mask slide down the palatopharyngeal curve along midline in the mouth until the front end of device was inserted into the hypopharyngeal cavity). When appropriate, re-inject the extracted gas into the laryngeal mask, connect the anesthesia machine to manually control breathing, and observe the chest rise. When the APL pressure valve is at 30cmH_2_O, when the airbag is pressed by hand, the thorax can be seen regularly undulating, and the end-tidal carbon dioxide waveform can be seen, which means that the laryngeal mask is well ventilated. Otherwise, the laryngeal mask needs to be adjusted (up–down/Chandy’s manoeuvre [10, 11], reversal method, inflating or deflating etc.) to obtain a satisfactory position. A maximum of three attempts were allowed and the number of attempts was recorded. Time for insertion of LMAs was from the time of taking the device in hand to the confirmation of proper placement of the device.

**Fig. 2:**
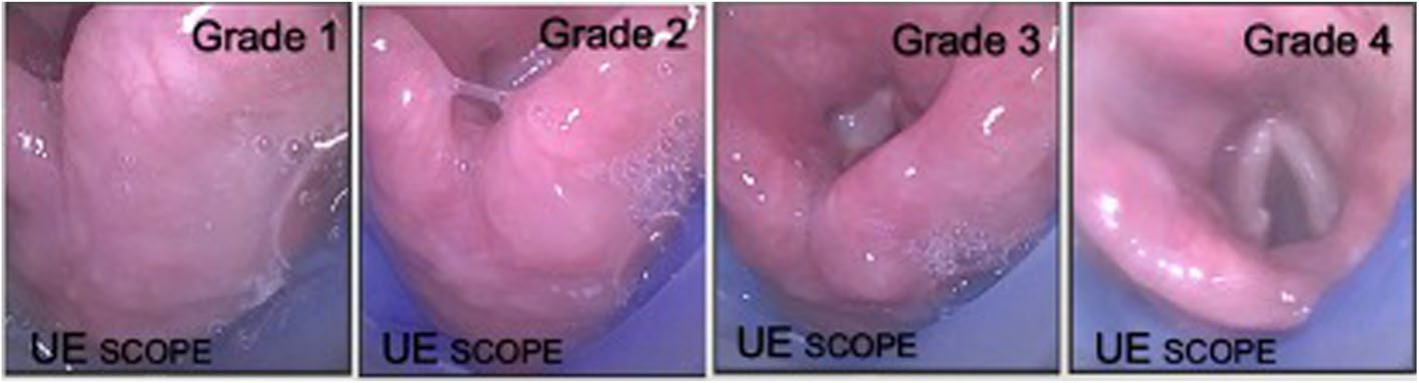
The classification by SaCoVLM. Grade 1: the lateral part of the right aryepiglottic fold and part of the laryngeal inlet and the ventilation was good. Grade 2: visualization of the bilateral aryepiglottic fold and part of the laryngeal inlet and the ventilation was good; Grade 3: visualization of all laryngeal inlet and partial glottis; Grade 4: full view of the glottis

Next, a fibreoptic evaluation of LMA placement was performed with a fibreoptic intubation scope, The glottis viewwas assessed and graded as follows [12]: Grade 1: no visualization of the glottis. Grade 2: no visualization of the glottis directly but can be found; Grade 3: partial view of the glottis; Grade 4: full view of the glottis; Additionally, the view on the screen was recorded. The classification of the glottis seen under the display of the SaCoVLM visual laryngeal mask (Fig. 2) [13].

**Fig. 3:**
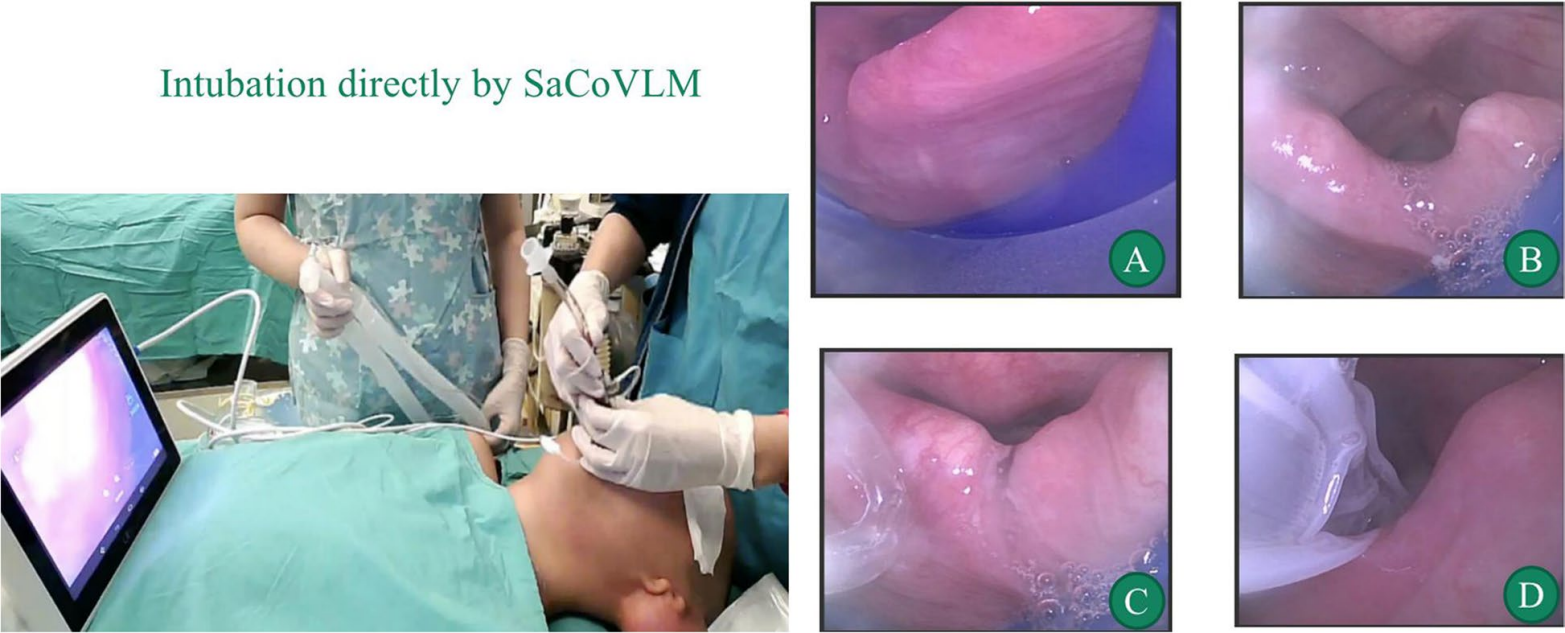
Intubation directly by SaCoVLM, the glottis was shown on the screen (**A**), and then inflated the cuff, the glottis would be more exposed (**B**), slided the tube through the intubation channel (**C**), rotated the tip of the tube to reach the openning of glottis, and then slided the tube into the tracheal (**D**)

SaCoVLM -guided intubation (Fig. 3): slide the endotracheal tube along the ventilation tube curve until meeting resistance and reaching the glottis; inflate the cuff (intracapsular pressure < 60 cmH2O), adjust the mask along the palatopharyngeal curve using the up-down maneuver to optimally expose the glottis, or rotated the tip of the tube to reach the openning of glottis, and then slided the tube into the tracheal.

Proper placement of the ETT was confirmed by the appearance of normal square wave capnogram and bilateral equal air entry. Time taken for intubation was recorded from the time of taking ETT in hand to the confirmation of proper placement of the ETT. During the procure, children were administered additional boluses of propofol (20–40 mg) to ensure adequate anesthetic depth.

After successful intubation, the LMA was removed using a stabilizing rod(a plain tracheal tube one size smaller was used as a removal guide). Time taken for removal of the device was recorded as the disconnection of the breathing circuit till ventilation successfully. The attempt was terminated and the attempt classified as “failure” if total time exceeded 300 s or SpO2 decreased to < 91% and the trachea would be intubated by direct laryngoscopy if the device was not achieved correctly after three attempts, fiberoptic intubation through the device was not successful after two attempts, or if the tracheal tube was dislodged during device removal.

Maintenance of anesthesia was realized using anesthetics (Oxygen+sevoflurane+propofol+remifentanil). Anesthetics were stopped at the end of surgery. Complications such as desaturation (SpO2 < 90%), regurgitation or aspiration, laryngospasm/bronchospasm, oropharyngeal or laryngeal trauma (blood staining of device/ETT) and hoarseness of voice were recorded. Patient follow-up was according to standard postoperative protocols at our institution.

### Data collection and statistical analysis

#### The primary variables

first-pass success rate of guided tracheal tube placement.

#### The secondary variables

glottic visualization grades, the first-attempt success rate of LMA placement, the time for LMA placement and time to endotracheal intubation as well as the time for LMA removal after successful intubation, the fiberoptic grade of laryngeal view, and the incidence 24 h complications after operation.

Data were recorded intraoperatively using a standardized data collection sheet and analyzed using Microsoft Excel Spreadsheet and the statistical software IBM SPSS software version 22 (SPSS Inc. Chicago, IL, USA). Study data are presented as means and standard deviation (SD) to describe continusous data and percentages for categorical data.

## Results

A total of 124 patients were enrolled in the study,including 94 males and 30 females with an average age of eight. The demographic data and clinical predictors of difficult airway, such as Mallampati score, mouth opening, and thyromental distance are shown in Table 1 and Table 2.

**Table 1:** Demographic data

Variable	SaCoVLM (*N* = 124)
Age (y)	8.0 ± 1.9
Weight (kg)	34.8 ± 9.9
Height (cm)	135.8 ± 11.9
Gender distribution (M:F)	94:30
Microtia	
Left/right	107 (86.3%)
Bilateral	17 (13.7%)
Anesthesia time (min)	194.5 ± 50.0

Values are absolute values (percent) and mean ± the standard deviation

**Table 2:** Clinical predictors of difficult airway

Results	SaCoVLM (*N* = 124)
Interincisor gap (cm)	4.5 ± 0.7
Thyromental distance (cm)	4.8 ± 0.7
Mallampati score n(%)	
1	67 (54%)
2	54 (43.5%)
3	3 (2.4%)
Upper lip bite test class n(%)	
I	75 (60.5%)
II	43 (34.7%)
III	6 (4.8%)
Hemifacial Microsomia n(%)	
0	61 (49.2%)
1	63 (50.8%)

Upper lip bite test class, Class I: lower incisors can hide mucosa of upper lip; Class II: I: lower incisors partially hide mucosa of upper lip; Class III: lower incisors unable to touch mucosa of upper lip; Values are absolute values (percent) and mean ± the standard deviation

The first-attempt success rate of SaCoVLM placement was 92.7% (95%CI = 1.03–1.13), therefore, a second attempt was required in 6.5 and 0.8% on the third attempt. All 9 patients underwent adjustments, including the up-down maneuver, increasing and reducing the amount of air, re-insertion and changing the laryngeal mask size. The average of SaCoVLM insertion time was 15 s.

The first-time success rate of SaCoVLM video laryngeal mask-guided intubation was 91.1% (95%CI = 1.04–1.14).113 cases were successfully intubated in the first time, 11 cases in the second time, The average time for successful endotracheal intubation was 30.9 ± 17.6 s, and The device were successfully removed in all patients without inadvertent extubation. The time for removal was 24.9 ± 9.3 s. Data regarding attempts on device placement and tracheal intubation through the devices are presented in Table 3.

**Table 3:** Outcomes of SaCoVLM video laryngeal mask-guided intubation

Result	Cases (%)/time(s)
Device placement attempts n(%)	
1	115 (92.7%) (95%CI = 1.03–1.13)
2	8 (6.5%)
3	1 (0.8%)
Insertion time (s)	15.7 ± 9.1
SaCoVLM size	
Size2.5	57 (46%)
Size3	67 (54%)
Intubation time (s)	30.9 ± 17.6
Withdrawl time (s)	24.9 ± 9.3
Tracheal tube placement attempts n(%)	
1	113 (91.1%)(95%CI = 1.04–1.14)
2	11 (8.9%)
Manoeuvres to optimize tracheal intubation n(%)	
Inflating the device	73 (58.9%)
Lifting the jaw	5 (4%)
Tracheal tube rotation	46 (37.1%)

Adjustmet of device placement: up-down manoeuvre; reinsert; chandy manoeuvre: consists of moving the SaCoVLM on a sagittal plane while ventilating to find the optimal position while lifting the device to provide a good seal

The glottis exposure classification on its screen is showed in Table 4. All patients were observed under fiberoptic intubation scope to classify the glottis again. The glottis can be well observed by SaCoVLM (78.2% cases could be observed glottis), the alignment between LMA and glottis is very good. The fiberoptic grade of laryngeal view was 100% glottis exposure. Three cases of unsuccessful intubation were excluded and related complications were not followed. The glottis exposure classification is showed in Table 4. All patients were observed under flexible intubation scope to classify the glottis again.

**Table 4:** Glottis exposure clsssification

	Grade1	Grade2	Grade3	Grade4
SaCoVLM (cases)	27 (21.8%)	36 (29%)	41 (33.1%)	20 (16.1%)
Flexible intubtation scope (cases)	0	15 (12.1%)	23 (18.5%)	86 (69.4%)

**SaCoVLM clsssification** is obtained by camera observation through the SaCoVLM visual channel: Grade 1: the lateral part of the right aryepiglottic fold and part of the laryngeal inlet, and the ventilation was good; Grade 2: the bilateral aryepiglottic fold and part of the laryngeal inlet, and the ventilation was good; Grade 3: Mainly partial view of all laryngeal inlet and posterior glottis; Grade 4: full view of the glottis;

**Flexible intubtation scope clsssification** is obtained by placing the flexible intubtation scope in the distal opening of the SaCoVLM vent. Grade 1: no visualization of the glottic entrance; Grade 2: visualization of glottis and the lingual surface of epi- glottis; Grade 3: visualization glottis and the laryngeal surface of the epiglottis. Grade 4: full visualization of the glottis only.

Postoperative complications are shown in Table 5. There was 3 case (2.4%) of bronchospasm after intubation but relieved by deepen anesthesia. Blood staining on the airway device was reported 31.5%. The incidence of postoperative sore throat at 2 h was 29, and 16.1% at 24 h, without dysphagia and hypoxia.

**Table 5:** Postoperative complications

Outcomes	SaCoVLM (*N* = 124)
Bronchospasm (%)	3 (2.4%)
Blood stains (%)	39 (31.5%)
sore throat at 2 h(%)	36 (29%)
sore throat at 24 h(%)	20 (16.1%)
hypoxia (%)	0
Dysphagia (%)	0

## Discussion

The first-attempt success rate of SaCoVLM insertion was 92.7% (95%CI = 1.03–1.13) in children, which confirms that the success rate of devices insertion is lower than that in adults with normal airway [13, 14], This may be due to the anatomical differences in children with microtia, which may existing a narrow laryngeal space. After manual adjustment [15] and reinsertion, the final success rate of device insertion can reach 100%.

The SaCoVLM contains a camera to capture the images of the glottis on a screen. We graded the images of 120 patients. Total track mask can visualize the glottis in 83% of patients [16], The glottis can be well observed by SaCoVLM (78.2% cases could be observed glottis), the alignment between LMA and glottis is very good. The fiberoptic grade of laryngeal view was 100% glottis exposure. The visualization of laryngeal mask can better observe the glottis position to facilitate the guidance of endotracheal intubation. Additional advantages with its use include a means for providing continuous oxygenation with a hands-free airway while potentially overcoming upper airway obstructions [17, 18]. All the children could display their partial or whole laryngeal inlet, which lays a foundation for tracheal intubation.

The first-attempt success rate of SaCoVLM video laryngeal mask-guided intubation was 91.1% (95%CI = 1.04–1.14). Previous findings that suggest blind intubations through supraglottic airway devices should not be performed in children as epiglottic downfolding may be present [19–21], so supplementary intubation instruments such as fibrobronchoscopy and bougie are sometimes required, however in SaCoVLM, intubation through video-guided can better locate the glottis, provide a more unobstructed airway, which decrease the equipment demand. When the inbuation process was not successful, we could use these methods, on one hand, the volume of air in the inflated mask cuff was increased to expand the space in the hypopharyngeal cavity and improve glottis exposure. The overinflated mask cuff further elevated the epiglottis root and improved glottis exposure. on the other hand, when the tip of the tube was lower than the interarytenoid notch and could not enter the glottis, we rotated the tip of tube and adjusted the relative position of the tube and glottis by up-down maneuver as well as push at left and right larynx. However, a more complete of glottis display correlates with a higher success rate of directly intubation. When the image displayed by the glottis is unsatisfactory, the exposure range of the glottis can be increased by increasing the amount of inflation and adjusting the depth of the laryngeal mask or rotating the tip of endotracheal tube.

Due to the need of surgery, all LMAs have been withdrawn. It should be noted that the tracheal tuber should not be taken out accidentally when withdrawing LMA. Another reasonable option may be to leave both the ETT and the SaCoVLM in place until the conclusion of the procedure.

The most common complication were sore throat (16.1%) and blood staining (31.5%), our rates were higher than those found in a previous study [22]. This may be explained not only by the association between small pharyngeal cavity volume and microtia for children [23], but also by the differences in children, which seemed to develop higher incidence of sore throat than adluts [24, 25].

In this study, the classification of SaCoVLM and FIS is quite different. The reason is that they observe in different sites. For FIS is through the laryngeal mask vent tube to observe the glottis at the end of the vent tube. the SaCoVLM camera is located on the right side of vent cuff, which is prone to the right side of the vent opening end. However this difference does not affect the intubaion process. It was not common that the camera heated up, maybe because the camera was not directly exposed, since the vent opening was a blind end, and we turned on the camera in advance to preheat and put it into the visual channel, and then inserted the LMA .

In this study, we excluded three patients. the reasons were as follows (1): after tracheal intubation, air leakage test was positive after inflating the cuff, we supposed there might be a damage on the tube, so we changed a new tube by cook bougie. (2) after tracheal intubation, air leakage test was negative before inflating the cuff, we guessed the tube size might too large for the boy, we changed the tube by using a cook bougie. (3) one boy suffered a pneumothorax after the surgery, which was a postoperative complications after costal cartilage graft performed procedure.

This study had several limitations. First, we did not study neonates or children with normal ears, maybe we could cooperate with other institution for further study. Second, the complications such as blood staining of these devices should be studied more detailed. Third, data were collected in an unblinded fashion, which may have introduced bias and age range was wide and associated with anatomical differences in children which may affect the results the scientific basis on which the sample size was determined, we will carry out comparative studies on different models for further study.

This single-center study as a initial study, aiming to lay a foundation for the later multi-center large sample study.

## Conclusion

The SaCoVLM video laryngeal mask-guided intubation is feasible in children, with a high success rate,could be a new promising device to guide intubation in airway management.

## Data Availability

The data that support the findings of this study are available from Chinese Academy of Medical Sciences & Peking Union Medical College but restrictions apply to the availability of these data, which were used under license for the current study, and so are not publicly available. Data are however available from the authors upon reasonable request and with permission of Chinese Academy of Medical Sciences & Peking Union Medical College.

## References

[1] Uezono S, Holzman RS, Goto T, Nakata Y, Nagata S, Morita S (2001). Prediction of difficult airway in school-aged patients with microtia. Paediatr Anaesth.

[2] Xu J, Chen K, Deng X, Wei L, Yang D, Wang Y (2020). Prediction of difficult laryngoscopy in school-aged patients with microtia. Minerva Anestesiol.

[3] Ghai B, Wig J (2009). Comparison of different techniques of laryngeal mask placement in children. Curr Opin Anaesthesiol.

[4] Krishna SG, Bryant JF, Tobias JD (2018). Management of the difficult airway in the pediatric patient. J Pediatr Intensive Care.

[5] Apfelbaum JL, Hagberg CA, Connis RT, Abdelmalak BB, Agarkar M, Dutton RP, Fiadjoe JE, Greif R, Klock PA, Mercier D, Myatra SN, O'Sullivan EP, Rosenblatt WH, Sorbello M, Tung A (2022). 2022 American Society of Anesthesiologists Practice Guidelines for Management of the Difficult Airway. Anesthesiology.

[6] Van Zundert AAJ, Gatt SP, Van Zundert TCRV, Kumar CM, Pandit JJ. Features of new vision-incorporated third-generation video laryngeal mask airways. J Clin Monit Comput. 2021. 10.1007/s10877-021-00780-3 Epub ahead of print. PMID: 34919170. 10.1007/s10877-021-00780-3 34919170

[7] Mallampati SR, Gatt SP, Gugino LD, Desai SP, Waraksa B, Freiberger D, Liu PL (1985). A clinical sign to predict difficult tracheal intubation: a prospective study. Can Anaesth Soc J.

[8] Lewis M, Keramati S, Benumof JL, Berry CC (1994). What is the best way to determine oropharyngeal classification and mandibular space length to predict difficult laryngoscopy?. Anesthesiology.

[9] Drage MP, Nunez J, Vaughan RS, Asai T (1996). Jaw thrusting as a clinical test to assess the adequate depth of anaesthesia for insertion of the laryngeal mask. Anaesthesia.

[10] Jagannathan N, Sohn LE, Sawardekar A, Chang E, Langen KE, Anderson K (2012). A randomised trial comparing the laryngeal mask airway supreme™ with the laryngeal mask airway unique™ in children. Anaesthesia.

[11] Zhi J, Deng XM, Yang D, Wen C, Xu WL, Wang L, Xu J (2016). Comparison of the air-Q with the air-Q intubating laryngeal airway as a conduit for Fiberoptic-guided tracheal intubation in children with ear deformity. Zhongguo Yi Xue Ke Xue Yuan Xue Bao.

[12] Brimacombe J, Berry A (1993). A proposed fiber-optic scoring system to standardize the assessment of laryngeal mask airway position. Anesth Analg.

[13] Yan CL, Chen Y, Sun P, Qv ZY, Zuo MZ (2022). Preliminary evaluation of SaCoVLM™ video laryngeal mask airway in airway management for general anesthesia. BMC Anesthesiol.

[14] Preece G, Ng I, Lee K, Mezzavia P, Krieser R, Williams DL, Stewart O, Segal R (2018). A randomised controlled trial comparing fibreoptic-guided tracheal intubation through two supraglottic devices: Ambu® AuraGain™ laryngeal mask and LMA® Fastrach™. Anaesth Intensive Care.

[15] Mishra N, Bharadwaj A (2020). Comparison of Fiberoptic-guided tracheal intubation through intubating laryngeal mask airway (ILMA) FastrachTM and Ambu® Aura-i™: a randomized clinical study. Cureus.

[16] Gómez-Ríos MÁ, Freire-Vila E, Casans-Francés R, Pita-Fernández S (2019). The TotaltrackTMvideo laryngeal mask: an evaluation in 300 patients. Anaesthesia.

[17] Van Zundert AA, Kumar CM, Van Zundert TC (2016). Malpositioning of supraglottic airway devices: preventive and corrective strategies. Br J Anaesth.

[18] Van Zundert AAJ, Kumar CM, Van Zundert TCRV, Gatt SP, Pandit JJ (2021). The case for a 3rd generation supraglottic airway device facilitating direct vision placement. J Clin Monit Comput.

[19] Asai T, Nagata A, Shingu K (2008). Awake tracheal intubation through the laryngeal mask in neonates with upper airway obstruction. Paediatr Anaesth.

[20] White MC, Cook TM, Stoddart PA (2009). A critique of elective pediatric supraglottic airway devices. Paediatr Anaesth.

[21] Yan CL, Zhang YQ, Chen Y, Qv ZY, Zuo MZ (2022). Comparison of SaCoVLM™ video laryngeal mask-guided intubation and i-gel combined with flexible bronchoscopy-guided intubation in airway management during general anesthesia: a non-inferiority study. BMC Anesthesiol.

[22] Gaddam M, Sethi S, Jain A, Saini V (2019). Comparison of air-QⓇ insertion techniques in pediatric patients with fiber-optic bronchoscopic assessment: a prospective randomized control trial. Korean J Anesthesiol.

[23] Xu J, Deng X, Yan F (2020). Airway management in children with hemifacial microsomia: a restropective study of 311 cases. BMC Anesthesiol.

[24] Mitobe Y, Yamaguchi Y, Baba Y, Yoshioka T, Nakagawa K, Itou T, Kurahashi K (2022). A literature review of factors related to postoperative sore throat. J Clin Med Res.

[25] Lautenbacher S, Peters JH, Heesen M, Scheel J, Kunz M (2017). Age changes in pain perception: a systematic-review and meta-analysis of age effects on pain and tolerance thresholds. Neurosci Biobehav Rev.

